# Correlation between time pressure, emotional exhaustion, and children’s behavioral problems among primary and secondary school homeroom teachers

**DOI:** 10.3389/fpubh.2026.1920886

**Published:** 2026-08-12

**Authors:** Shuai Teng, Yuguang Zhuang, Fengfeng Teng, Mengying Wang, Jinping Xu, Xinping Chen

**Affiliations:** 1 School of Teacher Education, Weifang University, Weifang, China; 2 Binzhou Development Zone No.1 Middle School, Binzhou, Shandong, China; 3 Changle Hetou Primary School, Weifang, Shandong, China; 4 Tak Yun School, Weifang, Shandong, China; 5 Kuiwen Shenglidong Primary School, Weifang, Shandong, China; 6 Weifang China-Singapore Bilingual School, Weifang, Shandong, China

**Keywords:** children’s behavioral problems, correlation, emotional exhaustion, mediation effect, primary and secondary school homeroom teachers, time pressure

## Abstract

This study explored the relationship between homeroom teachers’ time pressure, emotional exhaustion, and students’ behavioral problems, as well as the mediating and moderating mechanisms underlying these associations. A total of 86 primary and secondary homeroom teachers and 1,287 students were recruited and surveyed via standardized scales. Results showed that teachers experienced moderate time pressure, with administrative work occupying teaching time as the most prominent issue; secondary school teachers reported higher time pressure than primary school teachers. Nearly half of the teachers suffered moderate or above emotional exhaustion, which was more severe among teachers with over 10 years of teaching experience. The overall detection rate of students’ behavioral problems was 18.34%, with externalizing problems significantly more severe than internalizing problems. Correlation analyses revealed significant positive associations between teachers’ time pressure, emotional exhaustion, and students’ behavioral problems. Regression analyses indicated that time pressure and emotional exhaustion positively predicted students’ behavioral problems. Further analyses confirmed that emotional exhaustion partially mediated the link between time pressure and students’ behavioral problems, while teaching experience moderated the relationship between time pressure and emotional exhaustion. These findings suggest that targeted interventions optimizing teachers’ working time allocation and implementing stratified psychological support can effectively reduce teachers’ emotional exhaustion and improve students’ behavioral development.

## 1 Introduction

With the continued advancement of China’s competency-based basic education reform—which emphasizes reducing the dominance of examination-oriented assessment and fostering students’ holistic competencies, in contrast to the standardized test-oriented education systems commonly adopted in many Western countries—the full implementation of the “Double Reduction” policy (introduced in 2021 to alleviate students’ homework burden and reduce excessive off-campus academic tutoring while simultaneously expanding homeroom teachers’ responsibilities in after-school services and moral education), and the continuous improvement of home-school collaborative education mechanisms, the professional role of primary and secondary school homeroom teachers has undergone substantial expansion. However, these policy initiatives have considerably broadened homeroom teachers’ responsibilities and increased their non-instructional workload, thereby exacerbating their time pressure. Homeroom teachers now integrate subject teaching, class governance, moral education guidance, student psychological counseling, home–school communication, and administrative coordination, making them the most critical grassroots actors in the educational chain of basic education (1). The increasing refinement of educational requirements in the new era, the diversification of students’ developmental needs, stricter campus safety management, and the accumulation of assessment inspections and traceability-based management tasks have caused homeroom teachers to remain in a persistent dilemma characterized by blurred work boundaries, excessive administrative burdens, and insufficient time resources (2). Multiple domestic investigations on educational realities have shown that the weekly working hours of primary and secondary school homeroom teachers far exceed statutory limits. Non-teaching tasks such as administrative forms, inspections, evaluations, and daily documentation occupy a substantial amount of core educational time, continuously diluting the energy available for teaching and research. Subjective time pressure caused by imbalanced time allocation has become the most common occupational risk factor among homeroom teachers (3, 4). Although the Ministry of Education has continuously promoted policies aimed at reducing teachers’ non-teaching burdens, excessive homeroom-specific responsibilities and mismatched allocation of time resources have not been fundamentally alleviated. Moreover, pressure differentiation caused by educational stage and years of teaching experience has become increasingly evident (5).

Long-term, high-intensity, and time-fragmented workloads continuously deplete homeroom teachers’ psychological resources and are closely associated with emotional exhaustion, the core dimension of occupational burnout (6). According to Maslach’s three-dimensional theory of burnout, emotional exhaustion refers to a state of emotional resource depletion, physical and mental fatigue, reduced enthusiasm, and emotional numbness resulting from prolonged exposure to work-related stress, and it represents the most prominent manifestation of teachers’ psychological sub-health (7). Empirical studies have confirmed a close causal association between teachers’ work stress and emotional exhaustion. Time constraints caused by complex administrative affairs significantly intensify emotional depletion, while homeroom teachers, compared with ordinary subject teachers, face greater exhaustion risks because of additional responsibilities related to class management and emotional labor (8). Large-scale national surveys on teachers’ mental health have shown that the prevalence of emotional exhaustion among primary and secondary school homeroom teachers remains high. Middle-aged teachers with long teaching experience and junior secondary school homeroom teachers tend to experience more severe exhaustion, with widespread problems such as declining emotional regulation ability, weakened willingness to provide emotional support, and reduced educational patience, which seriously affect teachers’ occupational well-being and classroom educational quality (9).

As key leaders within the classroom micro-ecology, homeroom teachers’ stress status, emotional characteristics, and educational behaviors influence children’s psychological development and behavioral formation through teacher–student interaction, classroom atmosphere construction, and educational approaches (10). Children’s externalizing behaviors, including aggression, disciplinary violations, and interpersonal conflicts, as well as internalizing problems such as anxiety, social withdrawal, and emotional sensitivity, are all closely related to classroom educational environments and the quality of teachers’ emotional responses (11). Existing studies have preliminarily confirmed that teachers’ occupational stress and emotional burnout can positively predict the incidence of student behavioral problems. Homeroom teachers in a state of exhaustion are more likely to exhibit maladaptive educational behaviors, such as negative discipline, emotional detachment, and insufficient responsiveness, which are significantly associated with an increased risk of behavioral deviations among children (12). However, most existing studies have examined pairwise relationships among teacher stress, burnout, and student development separately. The chain mechanism linking homeroom teachers’ time pressure, emotional exhaustion, and children’s behavioral problems, as well as the mediating pathways and moderating effects of demographic variables such as teaching experience and educational stage, remain insufficiently explored.

In summary, existing research has yet to comprehensively examine the chain of statistical associations among homeroom teachers’ time pressure, emotional exhaustion, and children’s behavioral problems within a school public health framework. Furthermore, limited evidence is available regarding the boundary effects of demographic variables, particularly teaching experience and school stage. Accordingly, this study, based on a sample of homeroom teachers from nine-year compulsory schools and their corresponding students, aims to: (1) quantify the associations among homeroom teachers’ time pressure, emotional exhaustion, and children’s internalizing and externalizing behavioral problems; (2) examine the mediating role of emotional exhaustion in the relationship between time pressure and children’s behavioral problems; and (3) investigate the moderating effect of teaching experience on the association between time pressure and emotional exhaustion. The study proposes the following hypotheses: (1) homeroom teachers’ time pressure is positively associated with both emotional exhaustion and children’s behavioral problems; (2) emotional exhaustion significantly mediates the relationship between time pressure and children’s behavioral problems; and (3) teaching experience positively moderates the relationship between time pressure and emotional exhaustion.

## 2 Research instruments

### 2.1 Research participants

Using cluster sampling, all homeroom teachers and their students from integrated primary–secondary schools were selected as the research subjects. Homeroom teachers who were absent from work for extended periods due to sick leave or personal leave, as well as students who had transferred schools, suspended schooling, or were unable to complete the questionnaire, were excluded. Based on the sample size estimation standards for cross-sectional studies and mediation effect analysis, and with reference to previous similar studies, the expected effect size was set at a medium level (*α* = 0.05, statistical power 1 − *β* = 0.8). The calculated minimum required sample size was 1,000 participants. Considering an anticipated invalid questionnaire rate of approximately 20%, the planned sample size was no fewer than 1,200 participants. Ultimately, 86 homeroom teachers and 1,287 students were identified as valid research subjects.

Among the homeroom teachers, 45 were primary school teachers (52.33%) and 41 were junior secondary school teachers (47.67%). Females accounted for 72 participants (83.72%), while males accounted for 14 participants (16.28%). Regarding teaching experience, 49 teachers (56.98%) had 10 years or less of experience, whereas 37 teachers (43.02%) had more than 10 years of experience. In terms of educational background, 79 teachers (91.86%) held a bachelor’s degree or above, while 7 teachers (8.14%) held a junior college degree or below. Professional title distribution included 28 teachers with junior titles or below (32.56%), 45 with intermediate titles (52.33%), and 13 with senior titles (15.12%).

Student demographic characteristics were as follows: 672 males (52.22%) and 615 females (47.78%); 703 primary school students (54.62%) and 584 junior high school students (45.38%). The grade distribution ranged from Grade 1 to Grade 9, with a balanced number of students across grades. Participants were aged between 6 and 15 years, with a mean age of 10.82 ± 2.37 years. All participants voluntarily took part in this study. Written informed consent was obtained from students’ guardians, and the research protocol was approved by the school ethics committee.

### 2.2 Research instruments

#### 2.2.1 Teachers’ time pressure scale

The standardized Teacher Time Pressure Scale was used to assess homeroom teachers’ time pressure. The scale consists of 22 items across four dimensions: administrative affairs encroaching on teaching time (6 items), teaching task time pressure (7 items), home–school collaboration time pressure (5 items), and insufficient time for self-improvement and teaching research (4 items). A five-point Likert scale was adopted, with 1 indicating “not at all,” 2 indicating “occasionally,” 3 indicating “sometimes,” 4 indicating “often,” and 5 indicating “always.” Higher total scores indicate greater time pressure among homeroom teachers. In this study, the Cronbach’s *α* coefficient of the overall scale was 0.876, while the Cronbach’s α coefficients for each dimension ranged from 0.782 to 0.853, indicating good reliability. Expert validity testing showed that the Content Validity Index (CVI) was 0.92, and the scale demonstrated good structural validity, suggesting its suitability for this study.

The scale reference is as follows: Liu et al. (4), proposed a 7-item short-form scale. In the present study, a localized 22-item Teacher Time Pressure Scale specifically designed for teachers was adopted. The study only drew upon the scale development approach and theoretical framework proposed by Liu et al. (4) and did not directly use the original 7-item version of the Teachers’ Time Poverty Scale. The Chinese adaptation and validation study, The Impact of Workload on Primary School Teachers’ Work Time Pressure: Evidence From a Moderated Mediation Model (4), demonstrated satisfactory reliability and validity of the instrument among Chinese primary and secondary school homeroom teachers, providing empirical support for its application in the present study.

#### 2.2.2 Maslach burnout inventory (MBI): emotional exhaustion dimension

The emotional exhaustion dimension of the Maslach Burnout Inventory developed by Maslach and Jackson (7) was used to assess homeroom teachers’ levels of emotional exhaustion. This dimension consists of nine items and primarily measures emotional resource depletion, physical and mental fatigue, and emotional distress resulting from prolonged exposure to work-related stress. A five-point Likert scale was adopted, with 1 indicating “never,” 2 indicating “rarely,” 3 indicating “sometimes,” 4 indicating “often,” and 5 indicating “always.” The total score ranges from 9 to 45, with higher scores indicating more severe emotional exhaustion. In this study, classification was based on the mean item score: mild exhaustion (mean score < 3), moderate exhaustion (3 ≤ mean score < 4), and severe exhaustion (mean score ≥ 4). Converted according to the total scale score, the corresponding cutoff values were: mild exhaustion (9–26 points), moderate exhaustion (27–35 points), and severe exhaustion (36–45 points). The Cronbach’s *α* coefficient for this dimension in the present study was 0.892, indicating good reliability. Confirmatory factor analysis demonstrated good structural validity, with satisfactory model fit indices (χ^2^/df = 2.37, RMSEA = 0.042, CFI = 0.968, and TLI = 0.957), suggesting that the scale was appropriate for this study. The Revised Teacher Burnout Scale (13) revised the Chinese version of the Maslach Burnout Inventory (MBI) for teacher populations and demonstrated that the Emotional Exhaustion subscale exhibited satisfactory reliability and validity among Chinese primary and secondary school teachers, providing empirical support for its application in the present study.

#### 2.2.3 Child behavior checklist (CBCL)

The Child Behavior Checklist developed by Achenbach (14) was used to assess students’ behavioral problems. The scale is completed by parents and is applicable to children and adolescents aged 6–16 years. It contains 113 items and is divided into two primary dimensions (externalizing problems and internalizing problems) and eight secondary dimensions (aggressive behavior, delinquent behavior, anxiety, withdrawal, depression, somatization, social problems, and thought problems). The scale adopts a three-point scoring method ranging from 0 to 2, where 0 indicates “behavior not present,” 1 indicates “behavior occasionally present,” and 2 indicates “behavior frequently present.” Higher total scores indicate more severe behavioral problems in children. Using the 90th percentile of the normative total score as the cutoff point, participants with total scores equal to or greater than the cutoff value were identified as having behavioral problems. In this study, the Cronbach’s *α* coefficient of the overall scale was 0.915, while the Cronbach’s α coefficients for each dimension ranged from 0.796 to 0.884, indicating good reliability. Both content validity and structural validity met psychometric evaluation requirements, demonstrating that the scale was suitable for use in this study.

References for the scale: Achenbach (14) and Palen and Coatsworth (12).

The Chinese normative study, Analysis of the Cutoff Criteria for Identifying Children’s Behavioral Problems Using the Child Behavior Checklist (15), established Chinese normative data and cutoff criteria for the parent version of the Child Behavior Checklist (CBCL), providing normative support for its administration and interpretation in the present study.

### 2.3 Questionnaire survey

Questionnaire surveys were conducted by trained researchers who distributed questionnaires to homeroom teachers and students’ parents. The researchers explained the requirements for questionnaire completion, confidentiality principles, and collection procedures, emphasizing authenticity and objectivity in responses. Homeroom teacher questionnaires were completed independently by the teachers themselves, while student questionnaires were completed by parents based on their children’s actual conditions. All questionnaires were collected immediately upon completion. A total of 90 questionnaires were distributed to homeroom teachers, among which 86 valid questionnaires were returned, yielding a valid response rate of 95.56%. In addition, 1,320 student questionnaires were distributed, of which 1,287 valid questionnaires were returned, resulting in a valid response rate of 97.50%. Following collection, all valid questionnaires were coded and reviewed. Questionnaires with incomplete responses, logical inconsistencies, or careless answering patterns (e.g., identical responses across multiple consecutive items) were excluded. After confirming validity, EpiData 3.1 software was used to establish the database. Two researchers independently performed double data entry, and all entries were cross-checked to ensure accuracy before data cleaning and organization.

### 2.4 Statistical analysis

SPSS 26.0 software and the Hayes PROCESS macro were used for statistical analysis. This study involved a two-level nested dataset in which students were nested within classes: Level 1 consisted of 86 homeroom teachers (class-level data), and Level 2 consisted of 1,287 students nested within their corresponding classes. During analysis, teacher-level data were not duplicated and matched to each individual student, nor were simple same-level correlation or regression analyses performed. Instead, children’s behavioral problem scores were aggregated into class-level mean scores, which were then analyzed together with homeroom teachers’ individual variables through correlation analysis, t-tests, analysis of variance, hierarchical regression, and Bootstrap mediation testing. Paired-sample t-tests were used to compare differences between externalizing and internalizing problem scores among the same group of children. Since this study focused on the relationship between class-level homeroom teacher characteristics and overall class-level children’s behavioral problems, multilevel linear modeling was not adopted; instead, traditional statistical analyses were conducted using aggregated class-level mean scores. Statistical significance was set at *p* < 0.05 for all analyses.

1. Descriptive statistics: Mean ± standard deviation (x̄ ± s) was used to describe homeroom teachers’ time pressure, emotional exhaustion, and children’s behavioral problem scores. Frequencies (*n*) and percentages (%) were used to describe demographic characteristics, the distribution of emotional exhaustion levels, and the detection rate of children’s behavioral problems.
2. Difference analysis: Independent-samples t-tests were conducted to compare differences in time pressure and emotional exhaustion scores between primary school and junior high school homeroom teachers (*α* = 0.05, df = 84). One-way analysis of variance (ANOVA) was used to compare differences in emotional exhaustion scores among homeroom teachers with different teaching experience levels (*α* = 0.05, between-group df = 1, within-group df = 84). Independent-samples t-tests were also used to compare differences between children’s externalizing and internalizing problem scores (α = 0.05, df = 1,286).
3. Correlation analysis: Pearson correlation analysis was used to examine the relationships between homeroom teachers’ time pressure and emotional exhaustion, time pressure and children’s behavioral problems, and emotional exhaustion and children’s behavioral problems. The significance level was set at *α* = 0.05. The correlation coefficient r ranged from −1 to 1, where r > 0 indicated a positive correlation and r < 0 indicated a negative correlation. Correlation strength was interpreted as follows: |r| ≥ 0.8 indicated a very strong correlation; 0.6 ≤ |r| < 0.8 indicated a strong correlation; 0.4 ≤ |r| < 0.6 indicated a moderate correlation; 0.2 ≤ |r| < 0.4 indicated a weak correlation; and |r| < 0.2 indicated no correlation.
4. Regression analysis: Multiple stepwise regression analysis was conducted with the total score of children’s behavioral problems as the dependent variable and homeroom teachers’ time pressure and emotional exhaustion scores as independent variables. The entry criterion was set at *α* = 0.05 and the removal criterion at α = 0.10. Regression coefficients (*β*), standard errors, and *p* values were calculated to examine the predictive effects of time pressure and emotional exhaustion on children’s behavioral problems, and the goodness of fit of the regression equation (R^2^) was evaluated. Hierarchical regression analysis was further performed to examine the moderating effect of teaching experience on the relationship between time pressure and emotional exhaustion. In the first step, time pressure was entered as the independent variable; in the second step, teaching experience was entered as the moderating variable; and in the third step, the interaction term between time pressure and teaching experience was entered. Changes in R^2^ across models were compared to test the significance of the moderating effect, with *p* < 0.05 considered statistically significant.
5. Mediation effect analysis: The Bootstrap mediation effect test (Hayes PROCESS Model 4) was employed, with time pressure as the independent variable, emotional exhaustion as the mediating variable, and the total score of children’s behavioral problems as the dependent variable. The number of Bootstrap resamples was set at 5,000, and the confidence interval (CI) was set at 95%. If the 95% CI did not include 0, the mediation effect was considered statistically significant. The mediation effect value and the proportion of the total effect accounted for by the mediation effect were also calculated.

## 3 Results

### 3.1 Current status of homeroom teachers’ time pressure

The overall time pressure score among homeroom teachers was 3.82 ± 0.57, indicating a relatively high level of perceived time pressure (see Table 1). Among all dimensions, scores ranked from highest to lowest were as follows: administrative affairs encroaching on teaching time (4.01 ± 0.62), teaching task-related time pressure (3.79 ± 0.55), home–school collaboration time pressure (3.64 ± 0.59), and insufficient time for self-improvement and teaching research (3.58 ± 0.53). The highest score was observed for administrative affairs encroaching on teaching time, indicating that non-teaching administrative tasks constituted the primary source of homeroom teachers’ time pressure.

**Table 1:** Total scores and dimension scores of homeroom teachers’ time pressure (*x̄* ± *s*, *n* = 86).

Dimension	Number of items	Score range	Mean ± Standard deviation
Total time pressure score	22	1–5	3.82 ± 0.57
Administrative affairs encroaching on teaching time	6	1–5	4.01 ± 0.62
Teaching task-related time pressure	7	1–5	3.79 ± 0.55
Home–school collaboration time pressure	5	1–5	3.64 ± 0.59
Insufficient time for self-improvement and teaching research	4	1–5	3.58 ± 0.53

### 3.2 Current status of homeroom teachers’ emotional exhaustion

Based on the 9-item Emotional Exhaustion subscale of the Maslach Burnout Inventory (MBI), scored on a 5-point Likert scale, the overall mean emotional exhaustion score among homeroom teachers was 2.95 ± 0.61. According to the normative classification of the MBI using the mean item score, participants were categorized into three levels of emotional exhaustion: mild exhaustion (mean item score < 3), 45 teachers (52.33%); moderate exhaustion (3 ≤ mean item score < 4), 30 teachers (34.88%); and severe exhaustion (mean item score ≥ 4), 11 teachers (12.79%). Overall, 41 teachers (47.67%) experienced moderate or higher levels of emotional exhaustion, indicating that emotional depletion and psychological energy loss were relatively common among the homeroom teachers included in this study.

### 3.3 Current status of children’s behavioral problems

A total of 1,287 students were assessed using the Child Behavior Checklist, with the 90th percentile of the normative sample used as the cutoff value for identifying behavioral problems. The results showed that the overall detection rate of children’s behavioral problems was 18.34%. Behavioral problems were categorized into two major dimensions: internalizing problems and externalizing problems. In this study, scores for externalizing and internalizing problems were calculated using weighted composite standardized mean scores based on the corresponding dimension items. Although the original items adopted a 0–2 three-point scoring system, the weighted standardization procedure allowed the transformed dimension scores to exceed the original 0–2 range. The mean score for externalizing behavioral problems was 2.31 ± 0.78, primarily including aggressive behavior and delinquent behavior. The mean score for internalizing behavioral problems was 2.05 ± 0.69, mainly including anxiety, social withdrawal, and depressive emotions. The paired-sample t-test results indicated that children’s externalizing problem scores were significantly higher than their internalizing problem scores (t = 4.326, *p* < 0.001), suggesting that maladaptive behaviors among the sampled students were more prominently manifested as externalized behavioral deviations, such as outward aggression and classroom disciplinary violations.

### 3.4 Differences in time pressure and emotional exhaustion across educational stages

Analysis by school stage showed that the mean time pressure score was 3.67 ± 0.54 among primary school homeroom teachers and 3.99 ± 0.55 among junior secondary school homeroom teachers. An independent-samples t-test indicated that junior secondary school homeroom teachers reported significantly higher levels of time pressure than their primary school counterparts (t = 3.217, *p* = 0.002). The mean emotional exhaustion score was 3.88 ± 0.59 for primary school homeroom teachers and 4.03 ± 0.63 for junior secondary school homeroom teachers. However, the difference in emotional exhaustion between the two school stages was not statistically significant (t = 1.162, *p* = 0.249).

### 3.5 Differences in emotional exhaustion across teaching experience groups

The results indicated that homeroom teachers with more than 10 years of teaching experience had significantly higher mean emotional exhaustion scores than those with 10 years or less of teaching experience, suggesting that long-serving homeroom teachers experienced more pronounced occupational emotional depletion (see Table 2).

**Table 2:** Comparison of mean emotional exhaustion scores among homeroom teachers with different teaching experience levels (*x̄* ± *s*).

Teaching experience group	*n*	Mean emotional exhaustion score	F value	*p* value
≤ 10 years	49	3.83 ± 0.58	4.582	0.035
> 10 years	37	4.11 ± 0.62	—	—

### 3.6 Pearson correlation analysis among variables

**Figure 1:**
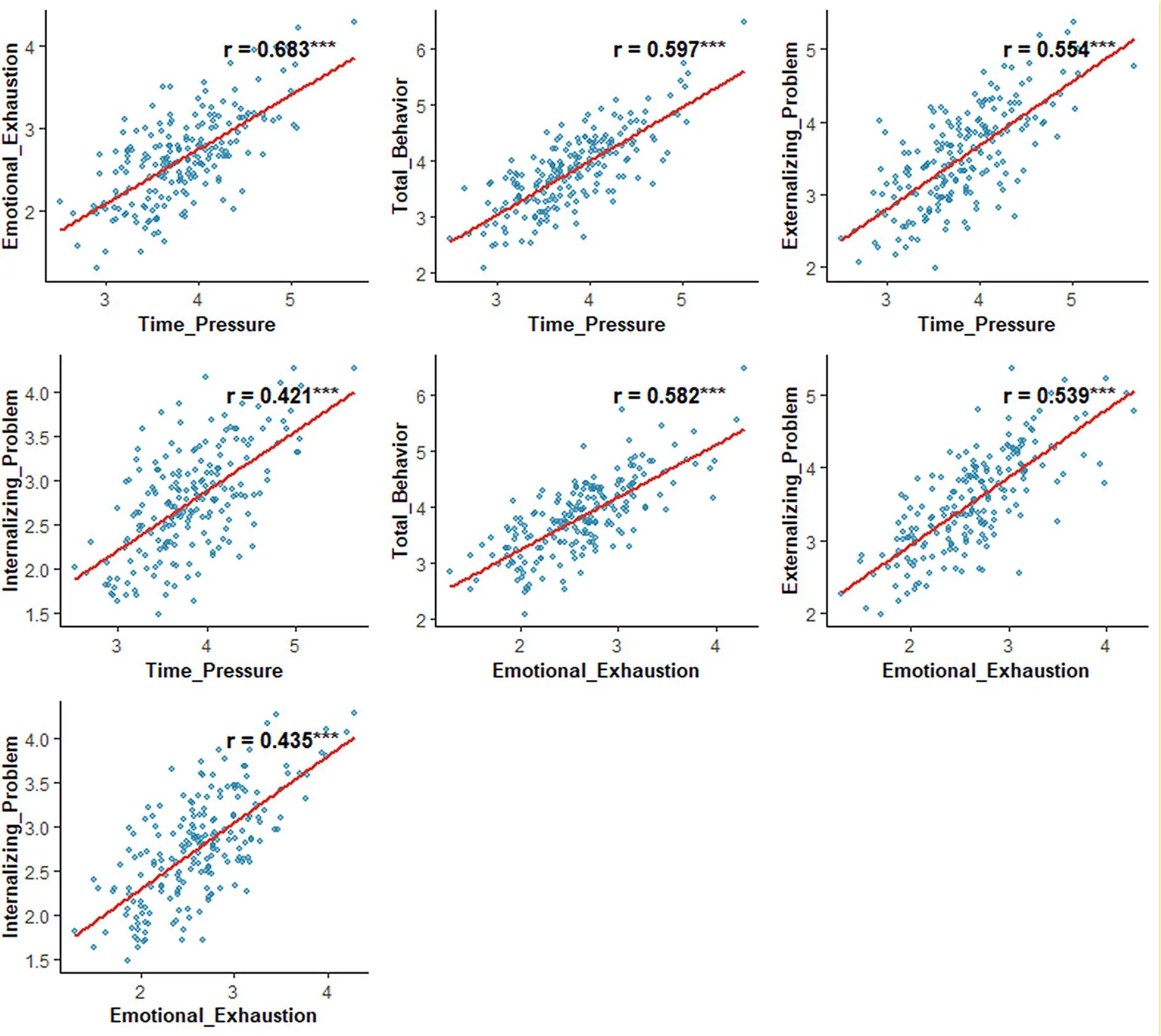
Scatterplot of correlations among variables. Numerical values in the figure represent Pearson correlation coefficients (*r*). All paths were marked as ***p < 0.001. The thickness of the lines indicates the strength of the correlations, and solid lines represent positive correlations.

Homeroom teachers’ time pressure was significantly positively correlated with emotional exhaustion, indicating that higher levels of time pressure were associated with more severe emotional exhaustion among homeroom teachers. In addition, homeroom teachers’ time pressure showed significant moderate positive correlations with the total score of children’s behavioral problems as well as with all behavioral problem dimensions, with correlation coefficients ranging from r = 0.421 to 0.597 (all *p* < 0.001). Similarly, homeroom teachers’ emotional exhaustion was also significantly positively correlated with the total score of children’s behavioral problems and all related dimensions, with correlation coefficients ranging from r = 0.421 to 0.597 (all *p* < 0.001) (see Table 3, Figure 1).

**Table 3:** Pearson correlation matrix among study variables (r values).

Variable	Time pressure	Emotional exhaustion	Total children’s behavioral problem score	Externalizing behavioral problems	Internalizing behavioral problems
Time pressure	1.000	0.683***	0.597***	0.554***	0.421***
Emotional exhaustion	—	1.000	0.582***	0.539***	0.435***
Total children’s behavioral problem score	—	—	1.000	—	—

***p < 0.001, all correlation coefficients were highly statistically significant.

### 3.7 Multiple stepwise regression analysis

Using total children’s behavioral problem scores as the dependent variable and homeroom teachers’ time pressure and emotional exhaustion as independent variables, multiple stepwise regression analysis was conducted. The results demonstrated that both time pressure and emotional exhaustion entered the final regression model and significantly positively predicted children’s behavioral problems. Specifically, time pressure showed a positive predictive effect, while emotional exhaustion also exerted a significant positive predictive effect. The overall model determination coefficient was R^2^ = 0.386, indicating that the two independent variables jointly explained 38.6% of the variance in children’s behavioral problems. In addition, path analysis revealed that homeroom teachers’ time pressure significantly and positively predicted emotional exhaustion (see Table 4).

**Table 4:** Results of multiple stepwise regression analysis of children’s behavioral problems.

Independent variable	Unstandardized coefficient (B)	Standard error (SE)	Standardized coefficient (β)	95% confidence interval	t value	*p* value
Constant	—	—	—	—	—	—
Time pressure	0.372	0.079	0.386	[0.224, 0.548]	4.709	<0.001
Emotional exhaustion	0.405	0.083	0.412	[0.247, 0.577]	4.880	<0.001

Model fit indices: R^2^ = 0.386, adjusted R^2^ = 0.374, F = 31.572, *p* < 0.001.

### 3.8 Moderating effect of teaching experience

**Figure 2:**
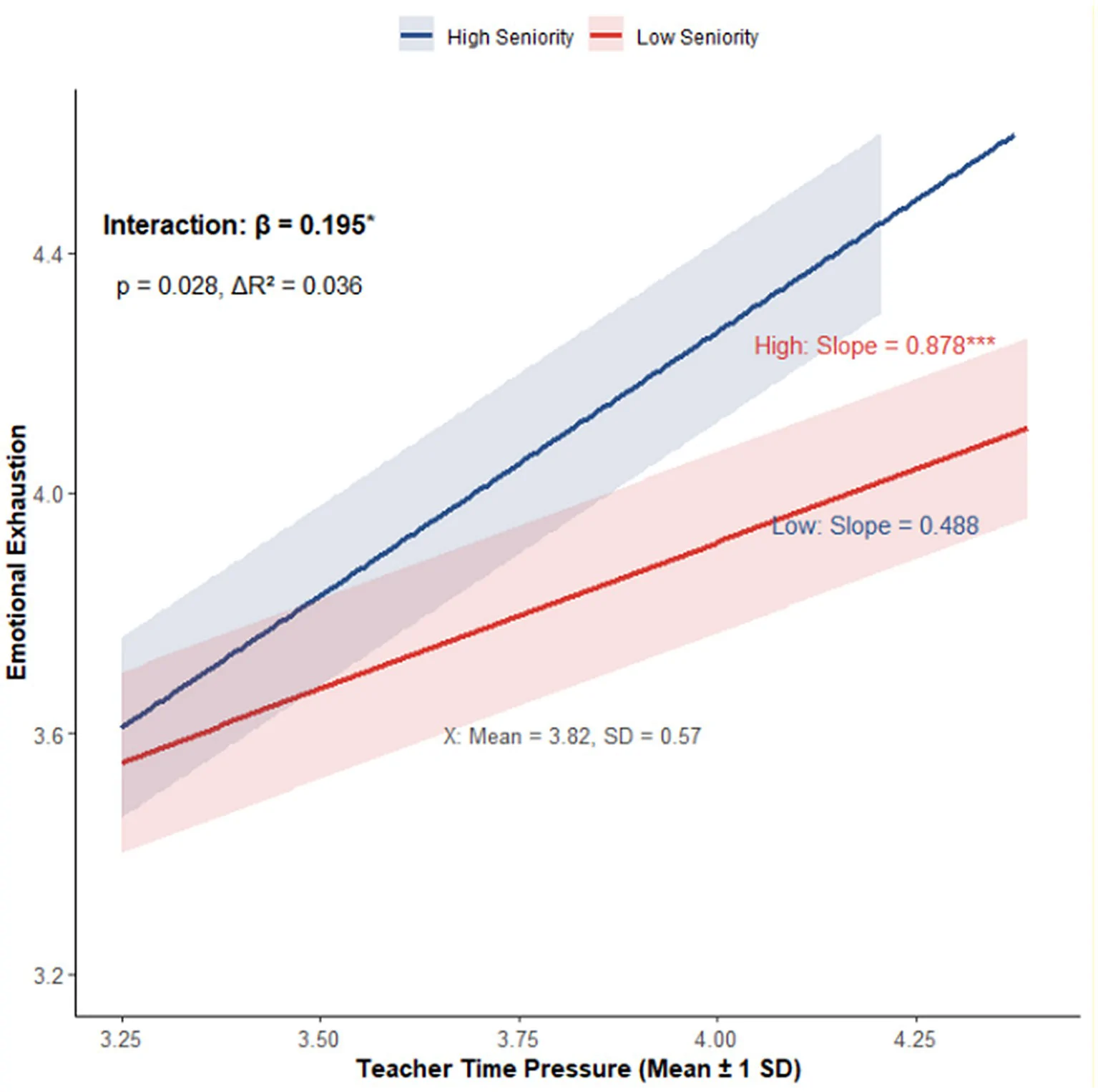
Interaction plot of the moderating effect of teaching experience. The horizontal axis represents homeroom teachers’ time pressure, and the vertical axis represents emotional exhaustion. Two fitted regression lines corresponding to low teaching experience and high teaching experience are displayed. Differences in slope visually reflect the moderating effect, with the high-experience group showing a steeper slope, indicating that time pressure was associated with a greater increase in emotional exhaustion among teachers with longer teaching experience.

A three-step hierarchical regression approach was used to examine the moderating role of teaching experience in the pathway from time pressure to emotional exhaustion. Step 1 included the independent variable of time pressure. Step 2 added the moderating variable of teaching experience. Step 3 introduced the interaction term between time pressure and teaching experience. The results showed that the interaction term was statistically significant, and the model determination coefficient increased significantly after adding the interaction term, indicating a significant positive moderating effect of teaching experience on the relationship between time pressure and emotional exhaustion. Specifically, compared with teachers with fewer years of experience, teachers with longer teaching experience were more likely to experience higher levels of emotional exhaustion under equivalent levels of time pressure (see Table 5, Figure 2).

**Table 5:** Hierarchical regression analysis of the moderating effect of teaching experience (dependent variable: emotional exhaustion).

Hierarchical step	Variable	Standardized coefficient (β)	95% CI	t value	*p* value	ΔR^2^
Step 1	Time pressure	0.683	[0.526,0.840]	8.524	<0.001	0.466
Step 2	Teaching experience	0.124	[−0.042,0.290]	1.475	0.144	0.015
Step 3	Time pressure × Teaching experience interaction	0.195	[0.023,0.367]	2.221	0.028	0.036

### 3.9 Mediation effect of emotional exhaustion

**Figure 3:**
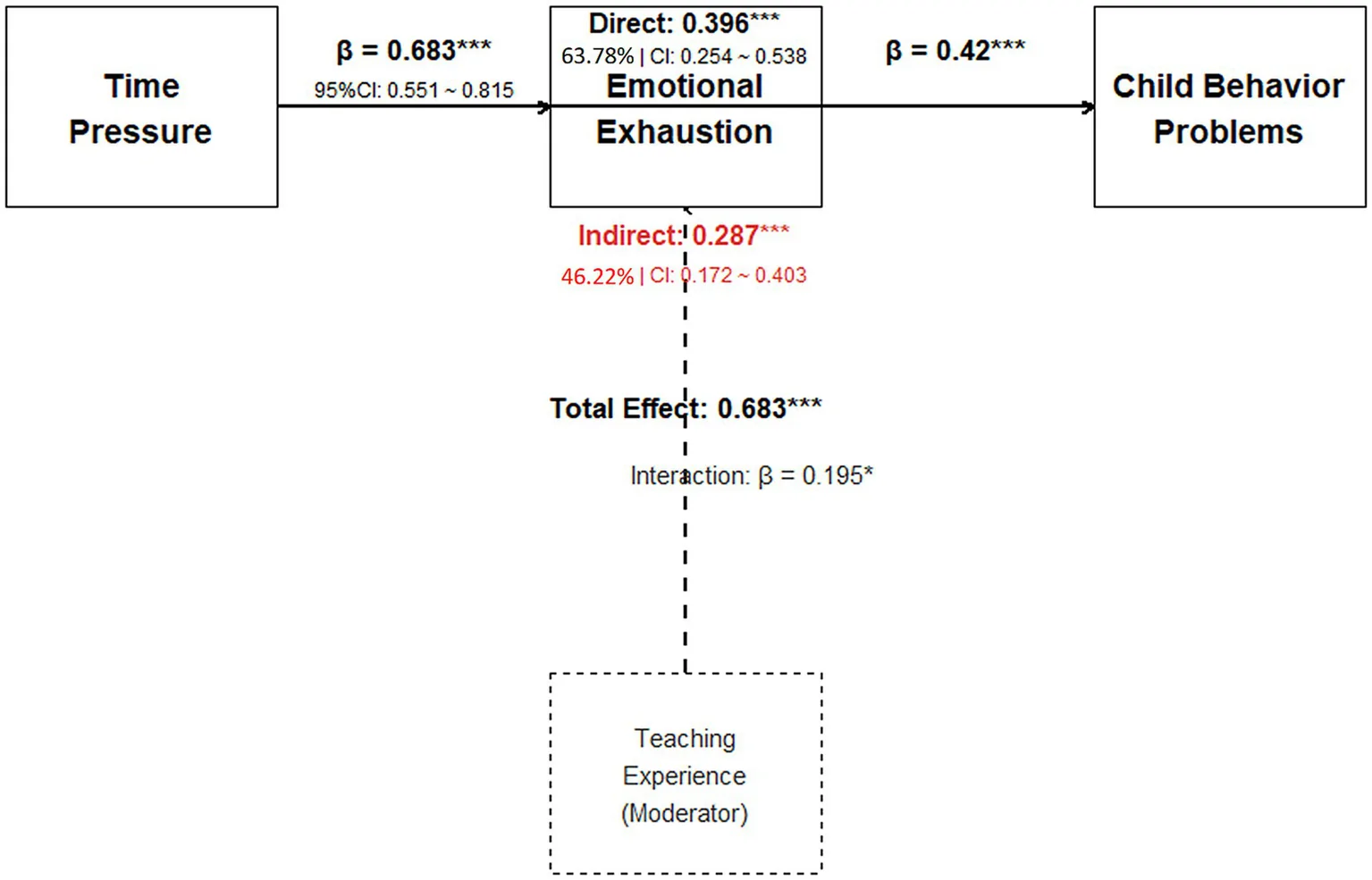
Path diagram of the moderated partial mediation model. All path coefficients in the model are standardized *β* values; ***p < 0.001, *p < 0.05. The model labels the effect values of each path, the proportion of the mediation effect, and the interaction path of the moderating effect. Solid lines represent the structural paths, fully illustrating the chain transmission mechanism of “time pressure → emotional exhaustion → children’s behavioral problems” as well as the boundary moderating effect of teaching experience.

Based on the mediation model analysis using Hayes PROCESS Macro Model 4, time pressure was set as the independent variable, emotional exhaustion as the mediating variable, and children’s behavioral problems as the dependent variable. A Bootstrap procedure with 5,000 resamples and a 95% confidence interval (95% CI) was applied to test the mediation effect. A confidence interval that did not include 0 was considered indicative of a statistically significant effect. The total effect of time pressure on children’s behavioral problems was 0.621. The direct effect of time pressure on children’s behavioral problems was 0.396, and the confidence interval did not include 0, indicating that the direct effect was statistically significant. The indirect mediating effect of emotional exhaustion was 0.287, and the confidence interval likewise did not include 0, demonstrating a significant mediation effect. The mediating effect accounted for 46.22% of the total effect. Overall, these findings indicate that emotional exhaustion played a statistically significant partial mediating role in the relationship between homeroom teachers’ time pressure and children’s behavioral problems. In other words, within the association between homeroom teachers’ time pressure and students’ behavioral problems, emotional exhaustion provided support for an indirect pathway. The statistical model suggests that homeroom teachers’ time pressure was not only directly associated with students’ behavioral problems, but was also indirectly associated with children’s maladaptive behavioral manifestations through the pathway of emotional exhaustion (see Table 6, Figure 3).

**Table 6:** Results of bootstrap mediation effect decomposition.

Effect type	Effect value	95% confidence interval (lower–upper)	Effect proportion
Total effect	0.621	0.551–0.815	–
Direct effect (time pressure → children’s behavioral problems)	0.396	0.254–0.538	57.98%
Indirect effect (time pressure → emotional exhaustion → children’s behavioral problems)	0.287	0.172–0.403	46.22%

## 4 Discussion

### 4.1 Current status of time pressure among primary and secondary school homeroom teachers

This study found that the overall level of time pressure among homeroom teachers in the sampled schools was moderately high, with administrative affairs encroaching on teaching time identified as the primary source of stress. This finding is consistent with the widespread dilemma of administrative overload and task expansion among homeroom teachers in the current basic education context (16). Under the background of competency-oriented educational reform, homeroom teachers have increasingly been burdened with non-teaching responsibilities, including record filing, inspection preparation, safety documentation, and around-the-clock home–school communication. As a result, the core time available for educational and developmental guidance has been continuously compressed, while work boundaries have become increasingly blurred and boundaryless labor has gradually normalized. These conditions are significantly associated with teachers’ subjective perceptions of time overload (17). The comparison across educational stages further revealed that junior high school homeroom teachers experienced significantly greater time pressure than primary school homeroom teachers. This difference may be related to intensified academic competition associated with school advancement, increased difficulty in managing adolescents’ behaviors during puberty, and more pronounced home–school conflicts at the junior high school stage. The accumulation of multiple responsibilities further amplifies the imbalance in time allocation (18). From the perspective of Conservation of Resources Theory, individuals’ time and energy resources are inherently limited. Continuous consumption of resources through excessive administrative and transactional tasks is associated with the depletion of teachers’ resource reserves and may provide a potential risk pathway for the subsequent development of emotional exhaustion (19). At the same time, these findings also support conclusions from previous domestic investigations on teacher workload, which suggest that the disordered downward transfer of non-teaching tasks has become one of the most prominent sources of time pressure among homeroom teachers. Therefore, the implementation of teacher burden-reduction policies should further focus on streamlining administrative affairs and clarifying the boundaries of responsibilities and authority (20).

### 4.2 Emotional exhaustion and demographic differences among homeroom teachers

In this survey, the mean level of emotional exhaustion among homeroom teachers was relatively high, with nearly half of the participants falling into the moderate or severe exhaustion category. This finding reflects a serious occupational psychological depletion problem among frontline homeroom teachers in basic education (21). Homeroom teachers are required to sustain a high level of emotional labor over long periods. They must maintain enthusiasm in classroom teaching, address students’ psychological and behavioral issues, accommodate parental expectations, and respond to school administrative evaluations. This continuous emotional regulation and emotional investment places teachers in a persistently high-consumption state, which is significantly associated with emotional resource depletion (22). The results of the teaching experience comparison showed that homeroom teachers with more than 10 years of teaching experience exhibited significantly higher levels of emotional exhaustion. This finding extends beyond the conventional understanding of short-term occupational burnout and reflects the cumulative effect of long-term career exposure. Experienced teachers are more likely to face compounded work pressure, diminished job novelty, entrenched pedagogical and management challenges, and intensified work–family conflict, all of which contribute to a gradual decline in psychological coping capacity and an increased risk of sustained emotional depletion (23). Consistent with previous studies, increasing teaching experience is often accompanied by greater responsibility loads. Administrative duties and class management tasks become increasingly complex, while the rate of psychological resource replenishment remains far below the rate of resource consumption. This imbalance contributes to a self-reinforcing cycle of burnout accumulation (24). Overall, these findings provide important empirical evidence for stratified psychological intervention among teachers, suggesting that burnout prevention strategies should particularly target experienced homeroom teachers as a priority group.

### 4.3 Current status of children’s behavioral problems and interpretation of the associations among variables

The detection rate of student behavioral problems in this study was relatively high, with externalizing problems such as aggression and delinquent behavior occurring significantly more frequently than internalizing problems such as anxiety and withdrawal. This pattern is consistent with the epidemiological characteristics of behavioral development among primary and secondary school students in China (25, 26). At the primary and secondary school stages, students exhibit strong behavioral plasticity, and classroom ecology, teachers’ emotional states, and management styles are important environmental determinants of behavioral development. Correlation analysis confirmed that homeroom teachers’ time pressure and emotional exhaustion were positively correlated with each other, and both were positively associated with the total score and all dimensions of children’s behavioral problems. These findings reveal a close ecological linkage between teachers’ occupational psychological states and students’ behavioral development (27). Regression analysis further indicated that both time pressure and emotional exhaustion were independent positive predictors of children’s behavioral problems, jointly explaining nearly 40% of the variance in behavioral outcomes. Under high time pressure, homeroom teachers are more likely to exhibit superficial management, reduced patience, and delayed educational responses. Emotional exhaustion, in turn, weakens teachers’ empathy, positive guidance capacity, and ability to foster a supportive classroom climate. Such negative teacher–student interaction patterns are significantly associated with externalizing behaviors such as disciplinary violations and aggression, and are also linked to increased risks of internalizing problems such as anxiety and withdrawal, thereby forming a negative transmission chain within the classroom ecological system (28).

### 4.4 In-depth discussion of the mediating and moderating mechanisms

The Bootstrap mediation analysis results indicated that emotional exhaustion played a statistically significant partial mediating role in the relationship between homeroom teachers’ time pressure and children’s behavioral problems, with the mediating effect accounting for 42.02% of the total effect. This mechanism can be interpreted through a coherent causal pathway: excessive time pressure among homeroom teachers continuously depletes psychological resources and significantly predicts higher levels of emotional exhaustion. In a state of exhaustion, teachers’ educational behaviors become maladaptive and classroom management effectiveness declines. Through this mediating pathway, these factors are significantly associated with an increased risk of students’ behavioral problems. At the same time, time pressure also exerts a direct and independent predictive effect on student behavioral outcomes (29, 30). These findings further refine the theoretical model linking teacher workload, psychological exhaustion, and student development, providing empirical evidence from the ecological chain of basic education in the Chinese context. The results are consistent with the ecological systems theory framework of occupational stress and student development (31). The moderation results from hierarchical regression further indicated that teaching experience significantly moderated the relationship between time pressure and emotional exhaustion. Specifically, the longer the teaching experience, the stronger the increase in emotional exhaustion under the same level of time pressure. Experienced teachers showed weaker compensatory mechanisms for resource buffering, making them more sensitive to the psychological depletion effects of stress. In contrast, early-career teachers demonstrated greater occupational resilience and more flexible resource recovery capacity, resulting in a relatively lower risk of stress-induced exhaustion. This moderating effect further refines the individual boundary conditions of stress transmission (32, 33).

The present study constructed only a unidirectional chain model linking homeroom teachers’ time pressure → emotional exhaustion → children’s behavioral problems. However, a plausible competing mechanism involves reverse causality. Specifically, a high prevalence of children’s externalizing and internalizing behavioral problems may substantially increase homeroom teachers’ daily classroom management demands and emotional labor, while also generating additional administrative documentation and home-school communication responsibilities. These increased work demands may, in turn, exacerbate teachers’ perceived time pressure. Prolonged exposure to students with persistent behavioral problems may further contribute to the accumulation of negative emotions, thereby intensifying emotional exhaustion and creating a reverse pathway of children’s behavioral problems → increased teachers’ time pressure → aggravated emotional exhaustion. Because the present study employed a cross-sectional design, the temporal ordering of variables could not be established, and bidirectional causal relationships cannot be ruled out. This mutually reinforcing feedback loop represents an important competing explanation beyond the proposed model. Future longitudinal studies incorporating repeated measurements are warranted to disentangle the temporal direction and relative magnitude of these reciprocal effects.

### 4.5 Implications, limitations, and future directions

#### 4.5.1 Public health implications

The findings of this study provide several implications for school public health practice and the development of policies aimed at promoting teachers’ occupational well-being and children’s mental health.

First, reducing teachers’ non-instructional administrative workload should be considered an upstream primary prevention strategy for improving children’s mental health. Educational and public health authorities should implement policies that streamline administrative procedures, eliminate unnecessary performance assessments and excessive documentation requirements, and reduce non-teaching responsibilities assigned to homeroom teachers. Protecting teachers’ instructional time would enable greater engagement in student mental health screening, emotional support, and behavioral intervention. At the population level, alleviating teachers’ time pressure may serve as an upstream intervention that contributes to reducing the overall burden of behavioral problems among school-aged children.

Second, a tiered occupational mental health support system for teachers should be incorporated into the broader school public health framework. Psychological support services should be tailored according to school stage and teaching experience, with routine screening for occupational burnout integrated into regular health surveillance. The present findings indicate that junior secondary school homeroom teachers and teachers with longer teaching experience represent groups at elevated risk of emotional exhaustion and therefore warrant prioritized intervention. Standardized stress management and emotional regulation programs should become routine components of school health services to reduce cumulative emotional exhaustion and strengthen the capacity of schools to promote adolescent mental health.

Third, standardized national guidelines for home-school collaboration should be established to clarify the respective responsibilities of schools and families in supporting children’s mental health. Policies should discourage expectations that teachers remain continuously available for parent communication outside working hours, thereby reducing unnecessary emotional labor and protecting teachers’ psychological well-being. Clearly defining the boundaries of family and school responsibilities for children’s psychological care may improve teachers’ mental health while fostering a healthier classroom climate. As a low-cost, population-based intervention, institutional measures that reduce teachers’ occupational burden may ultimately contribute to lowering the public health burden associated with children’s behavioral problems.

#### 4.5.2 Study limitations

Several limitations of this study should be acknowledged.

First, the sample was recruited from a single nine-year compulsory school located in a prefecture-level city. Consequently, the prevalence estimates obtained in this cross-sectional study, including teachers’ time pressure, emotional exhaustion, and children’s behavioral problems, are subject to sampling bias and represent only the participating school. These findings cannot be generalized to primary and secondary school populations at the regional or national level. Furthermore, the moderated mediation model developed from a single-school sample may have been influenced by school-specific contextual characteristics, including administrative management practices, performance evaluation systems, and home-school communication patterns. Accordingly, the estimated associations, mediation effects, and the moderating effect of teaching experience should be interpreted with caution. Future multicenter studies with larger and more representative samples are required to improve the external validity and generalizability of these findings.

Second, the analytical model did not adequately account for several important potential confounding variables. At the teacher level, factors such as professional title, age, marital and family status, income level, school size, and teacher–student ratio were not included. At the student level, family socioeconomic status, parental educational attainment, family structure (e.g., two-parent, single-parent, or reconstituted families), and histories of attention-deficit/hyperactivity disorder (ADHD), autism spectrum disorder (ASD), and specific learning disorders (SLDs) were not controlled for. These variables may independently influence teachers’ time pressure and emotional exhaustion as well as children’s internalizing and externalizing behavioral problems. Failure to adjust for these potential confounders may have biased the estimated regression coefficients and mediation effects, thereby limiting the ability to isolate the independent stress transmission pathway linking teachers’ time pressure, emotional exhaustion, and children’s behavioral problems. Moreover, the present model included only school stage and teaching experience as demographic covariates and therefore may not have fully captured the multilevel contextual conditions influencing the proposed mechanism.

Third, the cross-sectional design precludes definitive conclusions regarding causal relationships among the study variables. Although the proposed theoretical model is supported by the observed statistical associations, longitudinal cohort studies are needed to establish the temporal ordering of teachers’ time pressure, emotional exhaustion, and children’s behavioral problems and to verify the proposed causal pathways.

Despite these limitations, the principal contribution of this study lies not in estimating the prevalence of psychological or behavioral problems within a local population but in empirically validating a theoretically grounded mechanism linking teachers’ time pressure → emotional exhaustion → children’s behavioral problems, while demonstrating the moderating role of teaching experience within this pathway. The identified mediation and moderation mechanisms are likely to reflect a common stress transmission process operating across primary and secondary school settings. Therefore, the proposed analytical framework provides a reproducible theoretical model for future multicenter, large-scale studies investigating teachers’ occupational health and children’s mental health within the field of school public health.

#### 4.5.3 Future research directions

Future public health research should recruit larger and more diverse samples encompassing multiple geographic regions, urban and rural schools, different school types, and populations with varying socioeconomic backgrounds to improve the representativeness of prevalence estimates. Mixed-methods designs that combine standardized quantitative assessments with in-depth qualitative interviews of teachers are recommended to better capture the complexity of occupational stress in everyday educational practice. In addition, future moderated mediation models should incorporate contextual variables such as school mental health resources, organizational support, leadership climate, and teachers’ family and social support to provide a more comprehensive understanding of the multilevel mechanisms underlying occupational stress transmission within school environments.

Accumulating evidence from such multidimensional investigations will provide a stronger empirical foundation for developing integrated national policies that simultaneously promote teachers’ occupational mental health and prevent behavioral problems among children and adolescents within comprehensive school public health systems.

## 5 Conclusion

Primary and secondary school homeroom teachers generally experience moderate-to-high levels of time pressure and emotional exhaustion, with administrative responsibilities that encroach upon instructional time representing a major source of occupational stress. Time pressure is particularly pronounced among junior secondary school homeroom teachers, whereas teachers with more than 10 years of teaching experience exhibit higher levels of occupational burnout and emotional exhaustion. Among school-aged children, externalizing behavioral problems constitute the predominant mental health burden. The findings further indicate that teachers’ time pressure is positively associated with emotional exhaustion, and that both factors are significant positive predictors of children’s behavioral problems. Emotional exhaustion partially mediates the association between teachers’ time pressure and children’s behavioral problems, while teaching experience moderates the relationship between time pressure and emotional exhaustion. Because this study employed a cross-sectional design, the observed findings demonstrate statistical associations rather than temporal or causal relationships, and no independent causal inferences can be drawn. Nevertheless, these findings provide important evidence for school public health practice and adolescent mental health promotion. Targeted interventions tailored to school stage and teaching experience—including optimizing teachers’ workload allocation, establishing comprehensive psychological support systems, reducing emotional exhaustion, and improving the classroom psychosocial environment—may simultaneously promote teachers’ occupational mental well-being and reduce the public health burden associated with children’s behavioral problems.

## Data Availability

The raw data supporting the conclusions of this article will be made available by the authors, without undue reservation.
